# Microarray gene expression of periosteum in spontaneous bone regeneration of mandibular segmental defects

**DOI:** 10.1038/s41598-017-13586-8

**Published:** 2017-10-19

**Authors:** Zheyi Li, Juli Pan, Jinling Ma, Zhen Zhang, Yuxing Bai

**Affiliations:** 1 Department of Orthodontics, School of Stomatology, Capital Medical University, Beijing, China; 2 Department of Oral & Maxillofacial-Head & Neck Oncology, Shanghai Key Laboratory of Stomatology, Shanghai Ninth People’s Hospital, Shanghai Jiaotong University School of Medicine, Shanghai, China; 3 School of Stomatology, Beijing Stomatological Hospital, Capital Medical University, Beijing, China

## Abstract

Spontaneous bone regeneration could occur to reestablish mandibular bony continuity in patients who underwent partial or total mandibulectomy for tumors with periosteum-preserving. However, scarce data is available related to the precise role of periosteum in this bone regeneration. Therefore we aimed to investigate the gene expression of periosteum that were involved in the mandibular bone regeneration. Mandibular segmental defects were created in six mini-pigs with periosteum preserved. The periosteum of defects and control site were harvested at 1 and 2 weeks. Gene ontology (GO) analysis showed that the mechanisms concerning immature wound healing were clearly up-regulated at week 1. In contrast, by week-2, the GO categories of skeletal development, ossification and bone mineralization were significantly over-represented at week-2 with several genes encoding cell differentiation, extracellular matrix formation, and anatomical structure development. Furthermore, Tgfβ/Bmp, Wnt and Notch signaling were all related to the osteogenic process in this study. Besides osteogenesis, genes related to angiogenesis and neurogenesis were also prominent at week-2. These findings revealed that the gene expression profile of the periosteum’s cells participating in bone regeneration varied in different time points, and numbers of candidate genes that differentially expressed during early healing stages of intramembranous bone regeneration were suggested.

## Introduction

The periosteum is a specialized connective tissue sheath that covers the external surface of all the bones except for sites of articulation and muscle attachment. From a structural perspective, the periosteum is a highly vascularized and innervated bi-layered membrane with an inner cambium layer containing skeletal progenitor cells and osteoblasts, and an outer fibrous layer containing fibroblasts dispersed in between collagen fibers^1,2^. It’s in the past decade that researchers have conducted studies on the regenerative potential of periosteum, although its extraordinary regenerative capacity have been discussed for one century. It’s shown in several studies that compared to that of bone marrow and other cell sources, the endogenous regenerative potential of periosteum is higher and periosteum is now recognized as playing a crucial role in bone repair and regeneration^3^.

Although the regenerative capacity of periosteum has been demonstrated by many studies, the utilization of periosteum as a regenerative tool has been highly underestimated, especially in oral maxillofacial surgery region. Surgeons have found that spontaneous bone regeneration could occur to reestablish mandibular bony continuity in patients who underwent partial or total mandibulectomy for tumors with periosteum-preserving^4,5^. This phenomenon is rare but attracts an increasing attention from doctors and gives an enlightening resolution for endogenous bone tissue engineering and mandibular reconstruction. In our previous work, we have established a mini-pig mandibular segmental defects model to study this type of bone regeneration and the periosteum. Our results demonstrated remarkable osteogenesis potential of periosteum which showed that the critical size defects (CSD) of mandibular segmental defect was 6-cm with intact periosteum and 2-cm when periosteum was removed from mini-pig^6^. Nevertheless, there is scarce data available in relation to the precise role of periosteum in this bone regeneration and mechanism of this bone regeneration.

Transcriptome analysis using microarray technology is an effective method making the characterization of broad biological processes, as well as specific genes, that are expressed in a given cell population possible. This technology has been applied to investigate the transcriptional profile and hence clarify the biological processes related to bone healing^7,8^ and guided bone regeneration (GBR) *in vivo* ^9,10^. These reports have evaluated the molecular processes of bone regeneration and have found it to be an intricate process involving coordinated interplay between all kinds of various cell types. However, most studies characterizing the molecular mechanisms involved in bone regeneration uses the long bone defects or fracture model that indicates endochondral osteogenesis mainly. The bone in craniofacial region, developing in a different way from many other bones is derived from neural crest stem cells so that it has different characteristics to bones from other parts of the body, especially in the nature of its periosteum and ossification pattern. As intramembranous and endochondral ossification differ in many aspects and few data, if any, is available in relation to the role of periosteum in bone regeneration at the molecular level, these represent the incompletion in our knowledge.

Therefore, the purpose of this study was to use microarray technology to investigate the gene expression of periosteum that are involved in the bone regeneration of periosteum-preserving mandibular segmental defects in a mini-pig model by analyzing the transcriptional profile at two time points (1-week and 2-week). This is a crucial period in the bone regeneration process characteristic of both early response mechanisms and the subsequent processes involved in the initiation of osteogenesis underneath the periosteum. We hope that this study can provide an insight into the molecular regulation mechanisms of periosteum that occur during bone regeneration following periosteum-preserving mandibulectomy in a mini-pig model and help us to find out molecular targets that could be used to improve the regenerative process.

## Material and Methods

The study protocol was submitted and approved by the Animal Care and Use Committee, Beijing, China. All experiments were performed in accordance with relevant guidelines and regulations.

### Subjects and surgical procedure

Six 18-month-old adult female Chinese mini-pigs were used for this experiment. After a couple of weeks of acclimatization at the relevant facility, the experimental surgery was carried out under general anesthesia in accordance with our previous work^6^.

The six animals were randomized in two groups, with three animals each (A, B).

**Figure 1:**
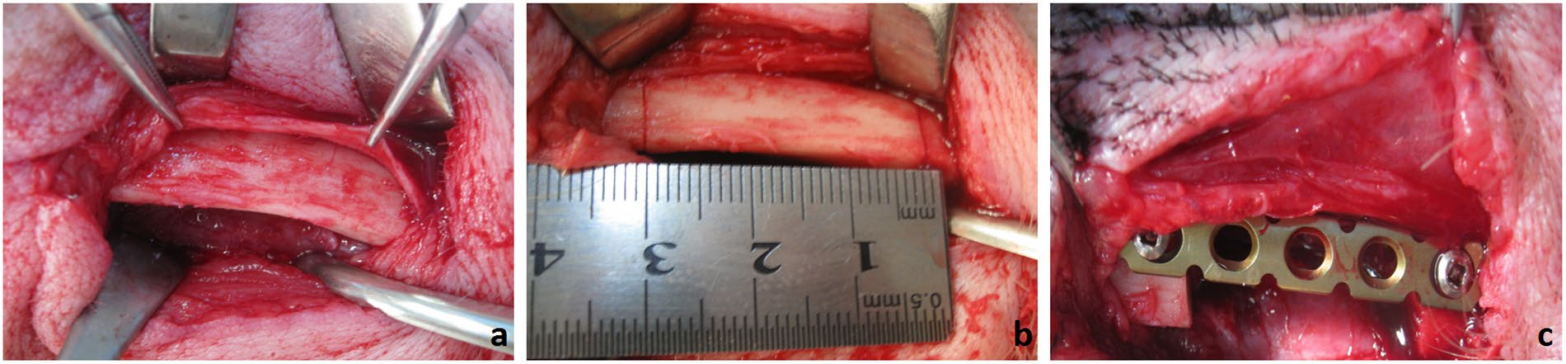
The surgical procedure. (**a**) The intact periosteum was bluntly and carefully detached from the cortical surface to expose the mandible. (**b**,**c**) 3-cm segmental mandible was resected with periosteum preserved, and a reconstruction plate was fixed across the defect.

Group A: The right mandible of each animal was selected for the experimental side while the left remained untreated. The premolar and the first molar of the experimental side had been extracted previously and the ridges had been allowed to heal for 2 months. In surgery, an extra-oral surgical approach was performed, the intact periosteum was bluntly and carefully detached from the cortical surfaces to expose the mandible and then 3-cm bone segment was resected. A reconstruction plate was fixed across the defect to maintain the mandible in the correct position. The periosteum was carefully and tightly sutured back in place and the periosteal envelop was preserved. The wound was closed in layers (Fig. 1). The healing period was 1 week. During sacrifice, the periosteum tissues of the defect site and untreated site were used for histological examination and microarray analysis.

Group B: In spite of the 2-week healing period, this group was treated in the same method.

### Histological examinations

At 1-week and 2-week, the periosteum tissues of defect and control site were carefully dissected. Part of tissue samples were then fixed with 4% paraformaldehyde for 12 hours at room temperature, and embedded in paraffin. Serial sections of 5μm thick sagittal slices were cut from the paraffin blocks with a microtome (SM2500E; Leica Microsystems, Wetzlar, Germany). The sections were stained with Mayer’s haematoxylin and eosin. Then they were observed using a light microscope (IDA-2000, Konghai Technology and Development Co., Beijing, China).

### Microarray analysis

The removed tissues were placed into liquid nitrogen, where they would freeze and be pulverized with a mortar and pestle and lysed in lysis buffer. The gene expression profiles of the samples were assessed using the Agilent Porcine Gene Expression (4 * 44 K, Design ID: 026440). Total RNA was quantified by the NanoDrop ND-2000 (Thermo Scientific) and the RNA integrity was evaluated by means of Agilent Bioanalyzer 2100 (Agilent Technologies). The sample labeling, microarray hybridization and washing were performed according to the manufacturer’s standard protocols. After washing, the arrays were scanned by the Agilent Scanner G2505C (Agilent Technologies).

### Data analysis

The images raw data was extracted by Feature Extraction software (Version 10.7.1.1, Agilent Technologies) and imported into Genespring (Agilent) for basic analysis. Differentially expressed genes were then identified through fold change as well as P value calculated with t-test. The threshold set for up- and down-regulated genes was a fold change ≥ 2.0 and a P value ≤ 0.05. Afterwards, functional analysis of these differentially expressed mRNAs was performed by utilizing the DAVID (The Database for Annotation, Visualization and Integrated Discovery) gene functional classification tool.

## Results

All surgeries were uneventful, therefore all animals could be used for gene expression analysis. And all samples featured good quality RNA, characterized by the RNA Integrity Number (RIN) ≥ 7 and 28S/18S band ratio ≥ 0.7^11^. All RNA samples were considered reliable and therefore utilized for transcriptomic analysis.

### Histology

**Figure 2:**
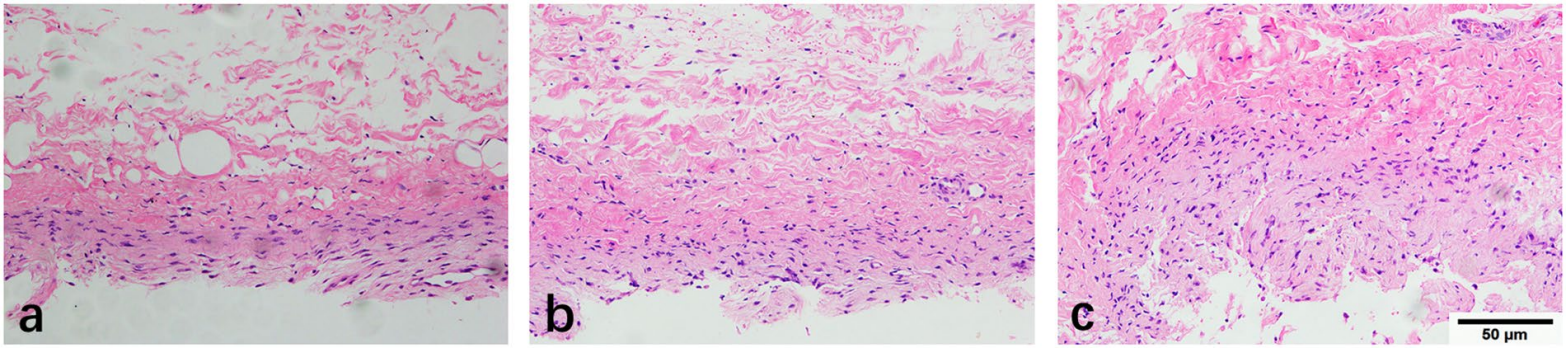
Histological examinations of periosteum of (**a**) control site, (**b**) defect site at week 1 and (**c**) defect site at week 2.

The images showed two different layers, outer fibrous layer and inner cambial layer of the periosteum. The outer fibrous layer contained fibroblasts dispersed in between collagen fiber and the inner cambium layer contained skeletal progenitor cells and osteoblasts. The cells of periosteum cambium layer at week-2 were much more active than that at week-1 and in control site. Furthermore, compared with the control site, the periosteum of defects site was thicker. And some adhesive muscle tissues were seen on fibrous layer of periosteum at week-2 (Fig. 2).

### Microarray analysis

**Figure 3:**
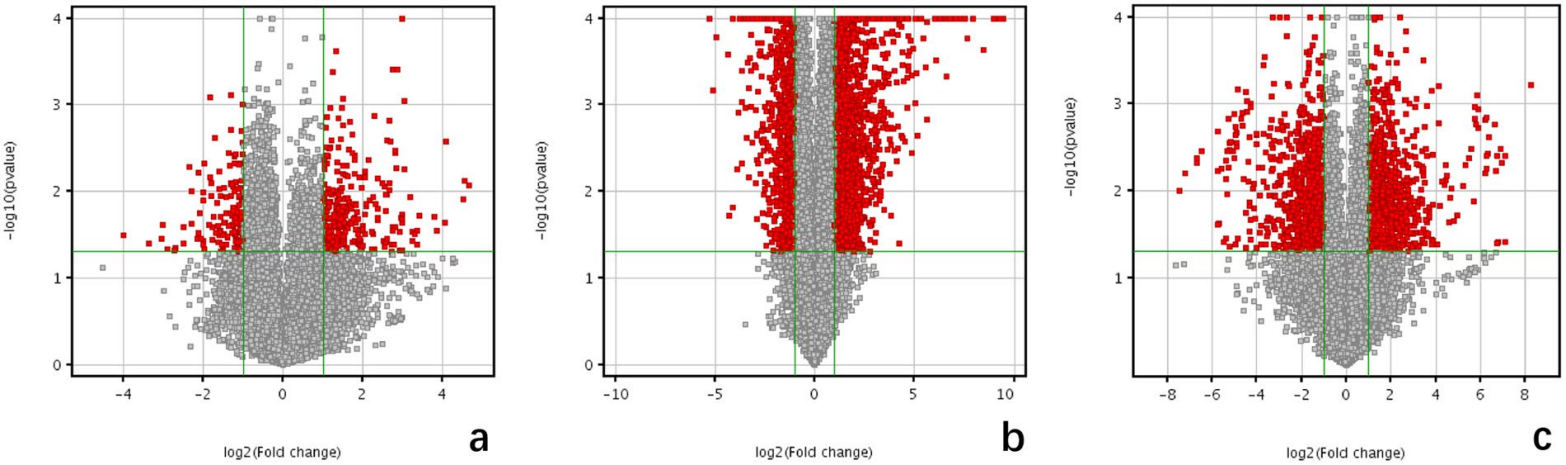
Volcano plots. (**a**) Week 1 vs. cs. (**b**) Week 2 vs. cs. (**c**) Week 2 vs. week 1. Red points indicate the mRNAs whose fold change ≥ 2.0 or ≤−2.0 and a P value ≤ 0.05.

A total of 439 genes had a statistically significant differential expression between periosteum of defects site and control site at week 1, with 297 being up-regulated and 142 down-regulated. A total of 1674 genes were differentially expressed between week 1 and week 2, with 891 being up-regulated at week 1 and 783 up-regulated at week 2. And a total of 1942 genes had a statistically significant differential expression between periosteum of defects site and control site at week 2, with 1065 being up-regulated and 877 down-regulated (Table 1). The volcano plots were created of these statistically significant genes (P < 0.05) (Fig. 3). To evaluate the functional significance of these differentially expressed genes, we used DAVID to identify the GO annotation terms that were notably enriched in these lists of genes (Tables 2–4).

**Table 1:** Numbers of statistically significant differentially regulated genes.

	Numbers of differentially significant genes		
	Increase	Decrease	Total
Week 1 vs. cs.	297	142	439
Week 2 vs. 1	783	891	1674
Week 2 vs. cs.	1065	877	1942

**Table 2:** Over-represented gene ontology (GO) terms (P < 0.05) in comparison between periosteum of defects site and control site at week 1, with number of genes indicated in parentheses.

Down-regulated at week 1	Up-regulated at week 1
**Cellular component**	**Cellular component**
GO:0005783~endoplasmic reticulum (12)	GO:0031224~intrinsic to membrane (96)
GO:0005759~mitochondrial matrix (5)	GO:0016021~integral to membrane (93)
	GO:0008305~integrin complex (7)
**Biological process**	**Biological process**
GO:0022610~biological adhesion (9)	GO:0009611~response to wounding (16)
GO:0034613~cellular protein localization (15)	GO:0045449~regulation of transcription (9)
GO:0030001~metal ion transport (12)	GO:0050817~coagulation (11)
GO:0006508~proteolysis (20)	GO:0007610~behavior (6)
GO:0006886~intracellular protein transport (7)	GO:0045449~regulation of transcription (7)
GO:0006979~response to oxidative stress (4)	GO:0030154~cell differentiation (26)
	GO:0006955~immune response (27)
	GO:0002526~acute inflammatory response (8)
**Molecular function**	**Molecular function**
GO:0030414~peptidase inhibitor activity (13)	GO:0046914~transition metal ion binding (42)
GO:0004857~enzyme inhibitor activity (14)	GO:0005529~sugar binding (10)
GO:0004175~endopeptidase activity (16)	GO:0020037~heme binding (10)
GO:0008270~zinc ion binding (21)

**Table 3:** Over-represented gene ontology (GO) terms (P < 0.05) in comparison between periosteum of defects site and control site at week 2, with number of genes indicated in parentheses.

Down-regulated at week 2	Up-regulated at week 2
**Cellular component**	Cellular component
GO:0031224~intrinsic to membrane (116)	GO:0044421~extracellular region part (35)
GO:0016021~integral to membrane (113)	GO:0005615~extracellular space (27)
GO:0044459~plasma membrane part (30)	GO:0042611~MHC protein complex (14)
GO:0005792~microsome (7)	GO:0005624~membrane fraction (7)
	GO:0005829~cytosol (10)
**Biological process**	**Biological process**
GO:0051252~regulation of RNA metabolic process (21)	GO:0006955~immune response (58)
GO:0006355~regulation of transcription, DNA-dependent (21)	GO:0048731~system development (41)
GO:0043408~regulation of MAPKKK cascade (9)	GO:0009611~response to wounding (19)
GO:0051952~regulation of amine transport (11)	GO:0007155~cell adhesion (20)
GO:0045449~regulation of transcription (27)	GO:0048513~organ development (19)
GO:0000165~MAPKKK cascade (13)	GO:0040007~growth (13)
GO:0042060~wound healing (5)	GO:0006954~inflammatory response (15)
GO:0048878~chemical homeostasis (5)	GO:0002504~antigen processing and presentation of peptide or polysaccharide antigen via MHC class II (6)
GO:0030001~metal ion transport (12)	GO:0019882~antigen processing and presentation (17)
GO:0006461~protein complex assembly (7)	GO:0022610~biological adhesion (20)
GO:0006812~cation transport (14)	GO:0001501~skeletal system development (12)
GO:0009988~cell-cell recognition (5)	GO:0060348~bone development (12)
GO:0030001~metal ion transport (12)	GO:0051302~regulation of cell division (5)
GO:0048584~positive regulation of response to stimulus (5)	GO:0030278~regulation of ossification (7)
GO:0042592~homeostatic process (8)	GO:0001525~angiogenesis (6)
GO:0050817~coagulation (4)	GO:0035265~organ growth (4)
	GO:0001503~ossification (4)
	GO:0016049~cell growth (7)
	GO:0030111~regulation of Wnt receptor signaling pathway (7)
	GO:0043393~regulation of protein binding (5)
	GO:0001944~vasculature development (3)
	GO:0030574~collagen catabolic process (3)
	GO:0016055~Wnt receptor signaling pathway (4)
	GO:0021700~developmental maturation (5)
	GO:0007179~transforming growth factor beta receptor signaling pathway (3)
	GO:0042136~neurotransmitter biosynthetic process (3)
	GO:0007218~neuropeptide signaling pathway (4)
Molecular function	**M**olecular function
GO:0046872~metal ion binding (78)	GO:0046914~transition metal ion binding (54)
GO:0005509~calcium ion binding (22)	GO:0030528~transcription regulator activity (24)
GO:0003700~transcription factor activity (18)	GO:0004175~endopeptidase activity (22)
GO:0005529~sugar binding (13)	GO:0008233~peptidase activity (27)
GO:0005516~calmodulin binding (5)	GO:0004950~chemokine receptor activity (8)
	GO:0019955~cytokine binding (12)
	GO:0030246~carbohydrate binding (18)
	GO:0008083~growth factor activity (9)
	GO:0004857~enzyme inhibitor activity (11)

**Table 4:** Over-represented gene ontology (GO) terms (P < 0.05) in comparison between periosteum of defects site at week 2 and 1, with number of genes indicated in parentheses.

Up-regulated at week 1	Up-regulated at week 2
**Cellular component**	**Cellular component**
GO:0031224~intrinsic to membrane (60)	GO:0005576~extracellular region (77)
GO:0043299~intracellular organelle (58)	GO:0031012~extracellular matrix (43)
GO:0005886~plasma membrane (31)	GO:0005783~endoplasmic reticulum (32)
Biological process	Biological process
GO:0006955~immune response (42)	GO:0032502~developmental process (70)
GO:0019882~antigen processing and presentation (15)	G0:0048856~anatomical structure development (65)
GO:0006954~inflammatory response (15)	GO:0048731~system development (54)
GO:0048002~antigen processing and presentation of peptide antigen (5)	GO:0048513~organ development (46)
GO:0009611~response to wounding (13)	GO:0030154~cell differentiation (26)
GO:0006952~defense response (16)	GO:005087~neurological system process (12)
GO:0002526~acute inflammatory response (8)	GO:0007155~cell adhesion (28)
GO:0040007~growth (21)	GO:0001501~skeletal system development (18)
GO:0007169~transmembrane receptor protein tyrosine kinase signaling pathway (12)	GO:0060348~bone development (12)
GO:0009967~positive regulation of signal transduction (17)	GO:0030500~regulation of bone mineralization (7)
GO:0007249~I-kB kinase/NF-kB cascade (5)	GO:0001503~ossification (6)
	GO:0030278~regulation of ossification (7)
	GO:0031214~biomineral formation (4)
	GO:0003018~vascular process in circulatory system (5)
	GO:0001944~vasculature development (4)
	GO:0001525~angiogenesis (4)
	GO:0007179~transforming growth factor beta receptor signaling pathway (9)
	GO:0007219~Notch signaling pathway (5)
	GO:0030509~Bmp signaling pathway (6)
	GO:0030111~regulation of Wnt receptor signaling pathway (5)
	GO:0016055~Wnt receptor signaling pathway (9)
	GO:0035023~ regulation of Rho protein signal transduction (9)
	GO:0007169~transmembrane receptor protein tyrosine kinase signaling pathway (3)
	GO:0046578~regulation of Ras protein signal transduction (3)
**Molecular function**	**Molecular function**
GO:0005509~calcium ion binding (16)	GO:0030246~carbohydrate binding (14)
GO:0005524~ATP binding (20)	GO:0005529~sugar binding (10)
GO:0032559~adenyl ribonucleotide binding (20)	GO:0005520~insulin-like growth factor binding (4)
GO:0043169~cation binding (46)	GO:0032403~protein complex binding (4)
GO:0046872~metal ion binding (45)	GO:0009975~cyclase activity (3)
	GO:0032403~protein complex binding (4)
	GO:0005518~collagen binding (2)
	GO:0022857~transmembrane transporter activity (16)

#### Selected GO terms significantly differentially expressed between periosteum of defects site and control site at week 1 and week 2

The results show that there was short of clear functional difference between periosteum of defects site and control site at week 1, genes associated with the inflammatory and immune response were over-expressed in the list. At week 2, functionally important GO categories were over-expressed in the list of genes that were up-regulated on defects site, including skeletal system development, bone development, regulation of ossification, Tgfβ and Wnt signaling. Furthermore, decreased intracellular metabolic activity was noted (Tables 2 and 3).

#### Selected GO terms significantly differentially expressed between week 1 and week 2

At week 1, in the cellular component category, genes related to intracellular localization in both the cytoplasm and plasma membrane were over-expressed in this list of genes. In keeping with this finding, the main molecular functions were associated with ion and ATP binding. As for biological processes, genes associated with the inflammatory and immune response were prevailing. The main signaling pathways over-expressed were positive regulation of signal transduction, transmembrane receptor protein tyrosine kinase signaling pathway, MAPKKK cascade and the immune process-related I-κB kinase/NF-kB cascade. At week 2, the cellular component category was distinguished by over-expression of genes localized within the extracellular matrix. In the aspect of molecular function, gene expression associated with the carbohydrate, sugar, protein, collagen and insulin-like growth factor binding was over-represented, as well as transmembrane transporter activity. The biological processes predominantly observed were cell differentiation and a variety of developmental processes, such as anatomical structure development and organ development, which are in accordance with a maturing wound. And genes related to skeletal development and ossification were highly over-expressed. In addition, this list was also characterized by the presence of genes related to angiogenesis and neurogenesis. Furthermore, gene expression related to the functionally relevant Tgfβ/Bmp, Wnt and Notch signaling pathways were over-expressed at week 2, as were genes related to the regulation of Ras and Rho protein signal transduction (Table 4).

### Functionally relevant gene expression

Genes related to osteogenesis and associated pathways regulated variously over the course of this study are shown in Tables 5–8.

**Table 5:** Gene members of the “Skeletal development” gene ontology group over-represented in the list of differentially expressed genes between week 1 and 2 (positive value indicates up-regulation at week 2).

Gene symbol	Gene name	Fold change
ACAN	Aggrecan 1	3.01
ACVR2B	Activin A receptor, type IIB	−2.02
BGLAP	Bone γ-Carboxyglutamate(gla) protein (osteocalcin)	8.32
BMP2	Bone morphogenetic protein 2	2.30
BMP4	Bone morphogenetic protein 4	2.30
COL11A2	Collagen, type XI,α2	4.08
COL1A2	Procollagen, type, 1α2	2.03
COL3A1	Collagen, type XIII, α1	3.81
COL5A2	Collagen, type V, α2	2.17
DMP1	Dentin matrix protein 1	13.31
FGFR1	Fibroblast growth factor receptor 1	2.44
HOXC4	Homeobox C4	6.39
MSX1	Msh homeobox 1	6.87
PTHLH	Parathyroid hormone-like hormone	10.76
PTGS2	Prostaglandin-endoperoxide synthase 2 (prostaglandin G/H synthase and cyclooxygenase)	6.02
RUNX2	Runt-related transcription factor 2	4.82
SPARCL1	SPARC-like 1 (hevin)	3.02
STC1	Stanniocalcin 1	3.38
TGFB3	Transforming growth factor, beta 3	5.53
THBS3	Thrombospondin 3	13.63
SMAD5	Smad homologue 5 (drosophila)	2.07
WNT5A	Wingless-type MMTV integration site family, member 5A	−2.08

**Table 6:** List of genes belonging to Tgfβ/Bmp signaling pathway that were significantly over expressed between week 1 and 2 (positive value indicates up-regulation at week 2).

Gene symbol	Gene name	Fold change
ACVR1	Activin A receptor, type I	2.21
BAMBI	BMP and activin membrane-bound inhibitor homolog (Xenopus laevis)	−3.17
BMP2	Bone morphogenetic protein 2	2.30
BMPR1A	Bone morphogenetic protein receptor, type IA	3.61
COL1A2	Procollagen, type, 1α2	2.03
DLX5	Homeobox protein DLX-5 isoform 2	3.84
GATA3	GATA binding protein 3	2.19
MAP3K1	Mitogen-activated protein kinase kinase kinase 1	9.53
SFRP2	Secreted frizzled-related protein 4	6.31
SMAD5	Smad homologue 5 (drosophila)	2.07
TGFB3	Transforming growth factor, beta 3	5.53
THBS3	Thrombospondin 3	13.63
ZFYVE9	Zinc finger, FYVE domain containing 9	2.12

**Table 7:** List of genes belonging to Wnt signaling pathway that were significantly over expressed between week 1 and 2 (positive value indicates up-regulation at week 2).

Gene symbol	Gene name	Fold change
APC2	Adenomatosis polyposis coli	4.2
AXIN2	AXIN2	−2.08
CPZ	Carboxypeptidase Z	8.3
CCND1	Cyclin D1	2.1
DKK1	dickkopf 1 homolog (Xenopus laevis)	2.3
DVL1	Dishevelled, dsh homologue 1 (Drosophila)	2.5
FZD1	Frizzled-1	2.1
FZD2	Frizzled-2	3.4
FZRB	Frizzled-related protein	9.7
LZTS2	Leucine zipper, putative tumor suppressor 2	2.1
LRP1	Prolow-density lipoprotein receptor-related protein 1	3.28
LRP5	Low-density lipoprotein receptor-related protein 5	2.05
WIF1	Wnt inhibitory factor 1	5.37
WISP2	WNT1-inducible-signaling pathway protein 2	6.15

**Table 8:** List of genes belonging to Notch signalling pathway that were significantly over expressed between week 1 and 2 (positive value indicates up-regulation at week 3).

Gene symbol	Gene name	Fold change
CFD	Complement factor D (adipsin)	3.12
DTX2	Deltex homolog 2 (Drosophila)	−2.61
FOXC1	Forkhead box C1	2.77
NOTCH4	Notch homolog 4 (Drosophila)	2.42
NRG1	Neuregulin 1	−2.64

#### Osteogenesis associated genes

Most genes were over-expressed at week 2, with few genes being over-expressed at week 1. The gene list includes lots of genes that have been extensively illustrated concerning their roles in osteogenesis, while other genes’ effects have not been elucidated and well-defined, thus warranting further studies both in terms of elucidating their biological impact in bone regeneration and revealing potential targets for therapeutic intervention. The well-recognized osteogenesis-related gene like osteocalcin and several collagens (Type V, XI, XIII) were among the extracellular matrix-related genes which were up-regulated at week 2, and a number of osteogenesis-related transcription factors were also obviously over-expressed, such as MSX1 and RUNX2. In general, the transcriptional profile was significative of a cellular behavior characteristic of osteogenic differentiation leading to the formation of the mineralized tissue (Table 5).

#### Tgfβ/Bmp signaling pathway associated genes

Thirteen Tgfβ/Bmp signaling-associated genes were regulated distinctly, and twelve of them were over-expressed at week 2. The over-expressed genes included the growth and differentiation factors themselves (BMP2), as well as receptors (ACVR1, BMPR1A, TGFBR3) and cytoplasmic signal transduction modulators (SMAD5, ZFYVE9) (Table 6).

#### Wnt signaling pathway associated genes

Fourteen Wnt pathway-related genes were differentially regulated, with the thirteen being up-regulated at the week 2. Genes related to the receptors FZD1, FZD2, LRP1 and LRP5, the cytoplasmic signal transduction molecules APC2 and DVL1 and the nuclear CCND1 (cycD) were up-regulated. Though AXIN2, one of the most powerful antagonists, was down-regulated at week 2, several antagonists of Wnt signaling are up-regulated (DKK1 and WIF1) and it is worth our attention (Table 7).

#### Notch signaling pathway associated genes

Relatively few Notch-associated genes were regulated distinctly. These genes were both up-regulated (CFD, FOXC1, NOTCH4) and down-regulated (DTX2, NGR1) at week 2 (Table 8).

## Discussion

Surgeons have been trying to reconstruct the mandible both functionally and aesthetically for more than a century, however, the reconstruction methods for mandibular defects used clinically are still problematic and mandibular defects reconstruction still remains a great challenge. Spontaneous bone regeneration following periosteum-preserving mandibulectomy is definitely beneficial and can be used in clinics as a reconstruction technique^4,5^. To re-establish the morphology as well as the function of lesion tissue by fully activating the healing potential of human body itself is a research direction in regenerative medicine. Spontaneous mandibular bone regeneration displays an enlightening resolution for mandibular defects reconstruction. The advantages of this bone regeneration include reduced biologic and economic costs, decreased morbidity and lower risk of complications as compared to other reconstruction methods, whereas a major deficiency of it is that the amount of spontaneously-generated new bone is sometimes unpredictable. Although we have already known that periosteum which has remarkable regenerative capacity is crucial in this bone regeneration procedure, little is known about the precise role of periosteum and mechanism of this periosteal-mediated bone regeneration. Therefore, this study focuses on the cellular and molecular regulation mechanisms of periosteum that occur during this bone regeneration, that may improve this method for better result of mandibular defects reconstruction.

The present study outlines the mechanisms of transcription with regard to periosteum in bone regeneration of a mandibular segmental defect of the mini-pig. This is a convincing as well as mature model for studying periosteum and its osteogenesis capacity in craniofacial region. Although the histological events with regard to periosteum and bone regeneration are thoroughly researched and intensively understood, it is the first time that the underlying molecular process related to periosteum in the regeneration of mandibular bone have been reported at a genome-wide level.

Bone regeneration, including four successive phases, is a complex and comprehensive process. The four stages consist of an initial inflammatory response and recruitment of skeletal progenitor cells, the formation of a cartilaginous callus, the replacement of cartilage by spongy bone and finally the remodeling of the immature bone into mature lamellar bone. The periosteum is the main contributor to this intricate process throughout all of these phases^12,13^. We compared transcriptional profiles of the two time points in this study so that the molecular and cellular mechanisms of periosteum mediating during early stage of bone regeneration can be established. Based on our previous study and some clinic cases, it is believed that 1-week to 2-week is a critical early bone regeneration period^9,10^. The highly specific and characteristic differences in gene expression of periosteum between 1-week and 2-week further validated the choice of the time points.

In our investigation, there was a statistically significant over-expression of intracellularly localized associated genes at 1-week, indicating early osteogenesis events. Conversely, the genes consistent with a maturing osteogenic wound, namely extracellular matrix-associated genes and differentiation-related genes, were predominantly expressed at week 2. In the respect of biological processes, the early periosteum-mediated bone regeneration at week 1 was related to an enhanced inflammatory and immune response. It has been suggested that the cellular inflammatory and immune response related to bone regeneration is unique as it is different from that found in the wound healing process of soft tissue and the modulation of this response may lead to improved bone regeneration outcomes^14–18^. Although the precise role of the inflammatory and immune response in the bone regeneration process is not fully clarified and further investigations are needed, modulation of the inflammatory process may represent a potential therapeutic target for improving bone regeneration. These findings are in agreement with an immature healing wound and are consistent with the results reporting on gene expression of bone fracture healing and GBR of cranial defects in rat models^7–10,19^. The transcriptional profile at week 2 is characteristic of a regenerating bone defect with the over-expression of large numbers of osteogenesis related mechanisms including ossification, skeletal system development and biomineral formation. Furthermore, cell differential and cell adhesion, genes related to bone extracellular matrix (DMP1, COL11A2, COL3A1, COL5A2), osteogenesis-related growth and differentiation factors (BMP2, TGFB3) as well as transcriptional regulators of osteoblast function (RUNX2) were all over-expressed at week-2. Significant osteogenic activity can be detected between week-1 and week-2 and this phenomenon is coordinated with the histological results that the periosteum of defects site at week-2 was thicker and the cells of cambium layer at week-2 were much more active. These findings are also in accordance with microarray studies of rat bone fracture healing and GBR of cranial defects^8–10,20^.

The osteogenesis mechanisms associated with the periosteum are accompanied by angiogenesis- and neurogenesis-related processes. Angiogenesis-related genes were over expressed at week 2 compared with week 1. This is in accordance with the finding of an increased angiogenesis-related gene expression in long bone fracture healing^8^ and calvarial GBR^9,10^ in the rat model. The up-regulation of the angiogenesis-related process is unsurprising as blood vessel formation is a well-understood requirement for bone regeneration or healing and periosteum provides a sufficient vascular supply to the newly-forming bone. Angiogenic factors are of great importance in promoting the process of regeneration^21,22^ and may represent another potential target for regulating the bone regeneration process. Neurogenesis-related genes were also over expressed when compared week 2 with week 1. This is consistent with the findings of other *in vivo* transcriptional studies, which have indicated a prominent neurogenesis-related gene expression during bone fracture healing and calvarial GBR in rat model^8,9,19^. However, the exact role of neurogenesis in bone healing or regeneration is not as well clarified, and nor is the impact of periosteum on neurogenesis-associated gene expression. Although this gene expression may indicate different rates of nerve fiber regeneration within the defect, recent studies of neuropeptide modulation of osteoblast function^23,24^ point out that neurogenesis-related genes may have a positive influence on promoting osteogenic processes in the craniofacial region. And some reports have indicated that there is a central nervous system control mechanism regulating the process of bone remodeling^25^. Given the over-expression of neurogenesis-related genes during periosteum-mediated bone regeneration, and more and more studies regarding the precise role of the nervous system in osteogenesis, the influence of neurogenesis during bone regeneration needs to be further studied.

Until now, little was known about the exact mechanisms that control the periosteum mediated bone formation. The present study elucidates the signaling pathways influencing the biological processes that are specific of the periosteum at early stage as well. At 1-week, the inflammatory and immune responses are followed by an up-regulation of genes related to the I-kB kinase/NF-kB signaling pathway. This is not astonishing for the sake that this pathway is not only intimately related to inflammation^26^ but also has been proved to have a deep effect on inflammation-induced bone loss^27,28^. At 2-week, the signaling pathways that were related to the up-regulation in osteogenesis-associated gene expression are Tgfβ/Bmp, Wnt and Notch. These signaling pathways have all been involved in bone formation and development^29–34^, and are comprehensively understood due to their important roles in the process of osteoblast differentiation^35–39^. As for bone regeneration, Bmp signaling appears to play a key role, with BMP2 being up-regulated in week-2. BMP2, famous for its powerful effect on skeletal development^30,40^, has been suspected to be an intrinsic initiator of bone regeneration for a long time^40^, indicating that BMP2 represents a primary regulator of periosteum-mediated bone regeneration. It is indicated that to achieve maximal therapeutic efficiency, the utilization of recombinant BMP2 in bone augmentation procedures may require the presence of the periosteum. It is found that in the injured periosteum, some members of the Wnt family are expressed^35,41^. Genetic studies in animal and human models indicate that the canonical Wnt pathway has a crucial role in embryonic bone, osteoblast differentiation and bone formation^41^. We found a positive regulation of the Wnt signaling pathway at week 2, and an up-regulation of genes encoding inhibitors of the canonical Wnt pathway (such as DKK1 and WIF1). However, unlike the Bmp target cells resident in the periosteum^42^, Wnt-responsive cells were indicated in other studies to be located mainly on the endosteal surface of injured bone, raising the possibility that during bone repair or regeneration, Bmp signaling and Wnt signaling act in different compartments within bone. Therefore, a Wnt-based therapy may indirectly promote periosteal-mediated bone regeneration while not directly target the periosteum. In addition, Ras and Rho signaling is up-regulated at 2-week as well. Both of these mechanisms are associated with osteoblast function, with Ras signaling is related to osteoblast differentiation^43^, while Rho being a downstream regulator of Wnt signaling^36^.

This *in vivo* transcriptional profiling study provides unprecedented insights into the multiple overlapping events involved in periosteum mediating mandibular bone regeneration by demonstrating the intricate interaction between several biological processes and signaling pathways. However, it has some limitations. First, only two time points were selected, so results can only be explained in the background of up/down regulation of genes between these two time points. Furthermore, the obtained tissue consists of varied cell types, making it impossible to attribute specific molecular mechanism to individual cell type and impractical to localize the source of the gene expression within the periosteum. Consequently, the findings of this study should be supplemented by *in situ* immunohistochemistry and hybridization in order to localize the protein and transcript expression within the periosteum, and have a broader and more precise picture of the mechanisms involved in periosteum mediated bone regeneration. And earlier and later healing periods should also be investigated by future studies.

Within the aforementioned limitation, this study has clarified the *in vivo* gene expression of periosteum that occurs during bone regeneration following periosteum-preserving mandibulectomy for the first time. The results will deepen our knowledge of the factors required for periosteum mediating bone healing or regeneration as well as provide potential targets for strategies aimed at promoting and enhancing regeneration in the regenerative process.
